# Promoting proactive bystander responses to racism and racial discrimination in primary schools: a mixed methods evaluation of the ‘Speak Out Against Racism’ program pilot

**DOI:** 10.1186/s12889-021-11469-2

**Published:** 2021-07-21

**Authors:** Naomi Priest, Oishee Alam, Mandy Truong, Rachel Sharples, Jacqueline Nelson, Kevin Dunn, Kate L. Francis, Yin Paradies, Anne Kavanagh

**Affiliations:** 1 Centre for Social Research and Methods, College of Arts & Social Sciences, Australian National University, RSSS Building, Canberra, 2601 Australia; 2 Population Health, Murdoch Children’s Research Institute, Royal Children’s Hospital, Melbourne, Victoria Australia; 3 School of Social Sciences & Psychology, Western Sydney University, Penrith South, New South Wales Australia; 4 Monash Nursing and Midwifery, Monash University, Clayton, Victoria Australia; 5 Menzies School of Health Research, Darwin, Northern Territory Australia; 6 Faculty of Arts and Social Sciences, University of Technology Sydney, Sydney, New South Wales Australia; 7 School of Humanities and Social Sciences, Faculty of Arts and Education, Deakin University, Burwood, Victoria Australia; 8 Centre for Health Equity, Melbourne School of Population and Global Health, University of Melbourne, Melbourne, Victoria Australia

**Keywords:** Racism, School-based intervention, Child health, Mixed-methods

## Abstract

**Background:**

Racism and racial discrimination are fundamental causes and determinants of health and health inequalities globally, with children and adolescents particularly vulnerable. Racial discrimination is a common stressor in the lives of many children and adolescents, with growing evidence of negative associations between racial discrimination and multiple domains of child and adolescent health. Addressing racism and racial discrimination must be core public health priorities, even more so among children and young people.

Schools are key settings in the lives of children and adolescents and become increasingly more important to identity formation. School communities, teachers and peers greatly influence children and adolescents’ beliefs about race and difference. Schools are therefore key sites for the delivery of population-based programs to reduce racism and promote proactive bystander behaviour and healthy resistance to racism among all children and adolescents as well as among the adults.

**Methods:**

This study examines the feasibility and acceptability of the ‘Speak Out Against Racism (SOAR)’ program, a whole of school, multi-level, multi-strategy program that aimed to promote effective bystander responses to racism and racial discrimination in primary schools. A mixed-methods, quasi-experimental design was used. Students in Years 5 and 6 (10–12 years) across six schools completed surveys pre- and post- intervention (*N* = 645; 52% female; 6% Indigenous, 10% Middle Eastern, African, Latinx or Pacific Islander, 21% Asian, 52% Anglo/European). Focus groups with students and interviews with staff collected qualitative data about their experiences of the program and their views about the program’s perceived need, implementation, impacts and suggested improvements.

**Results:**

Quantitative data showed student prosocial skills and teacher inter-racial climate improved in intervention schools compared to comparison schools. Qualitative data highlighted teacher attitudinal and behaviour change regarding racism, and student reduced interpersonal racial discrimination, improved peer prosocial norms, commitment to anti-racism, knowledge of proactive bystander responses and confidence and self-efficacy to intervene to address racism.

**Conclusions:**

This study provides quantitative evidence of the potential of the SOAR program to improve the prosocial skills of students and their perceptions of the inter-racial school climate provided by their teachers. This program also provided qualitative evidence of the potential to promote teacher and student attitudinal and behavioural change. Further refinement and testing of the program in a large scale implementation trial is recommended.

**Supplementary Information:**

The online version contains supplementary material available at 10.1186/s12889-021-11469-2.

## Background

Racism and racial discrimination are fundamental causes and determinants of health and health inequalities globally [1, 2]. Children and adolescents are particularly vulnerable to racism’s harms [3, 4]. Racism is an organised system of oppression that classifies and ranks social groups into ‘races’ and devalues, disempowers and differentially allocates power and resources to those considered inferior [5, 6]. Racial discrimination is the behavioural expression of racism by actions at individual or institutional levels [7]. Racism and racial discrimination have profound impacts on the lives of children and adolescents, their families and communities, shaping risks and opportunities and creating unjust, unnecessary, and preventable differences in health between social groups throughout the life-course and across generations [8, 9]. The COVID-19 pandemic and concurrent resurgence of the Black Lives Matter movement and attention to the public health emergency of racism around the world have heightened attention to the enduring social and structural injustices that are derived from racism as a system of oppression and of the critical need for action to address them [10].

Racial discrimination is a common stressor in the lives of many children and adolescents, with growing empirical evidence of negative associations between racial discrimination and multiple domains of child and adolescent health [4, 11–13]. Longitudinal studies have documented effects of racial discrimination on mental health [14, 15], substance use [16], cortisol dysregulation [17], allostatic load [18], epigenetic aging [19] and inflammation [20] among youth. Evidence also documents that impacts of racial discrimination are not limited to experiences where children and adolescents are direct targets of racism. Vicarious experiences of racial discrimination, including witnessing as a bystander or hearing about others’ experiences, are also associated with child and adolescent health outcomes including negative and positive dimensions of mental health and sleep duration, latency and quality [21, 22]. Concern about increasing societal discrimination is also associated with adolescent behavioural outcomes and depression [23]. These findings suggest that wide-ranging action and population-level interventions to promote societal anti-racism and bystander action by individuals are needed. Population-level health interventions are those that alter underlying conditions and determinants of health and health inequities in order to change the distribution of health risk across a population or large group of people [24]. If we are to optimise wellbeing for all children, adolescents and their adult caregivers, we must work towards eradicating racism and both direct and vicarious racial discrimination from the lives of children and adolescents [25]. Evidence-based population level programs that counter racism in education settings and that promote healthy resistance to racism among all children and adolescents as well as among the adults in their lives are identified as key priorities for child and adolescent health in a 2019 policy statement from the American Academy of Pediatrics [25]. Rigorous research that examines the impacts of such interventions on child and adolescent health remains an outstanding priority, with health equity unable to be achieved without addressing racism as a fundamental cause [25, 26].

The importance of teaching children and adolescents to recognise and reject racism and racial discrimination is also supported by developmental science research that shows childhood and adolescence are foundational to the development of prosocial and intergroup attitudes, beliefs and behaviours as well as to identity formation [27–29]. However, while doing so has many potential benefits for population health equity and for individuals and their families, as with anti-racism programs more broadly, such programs also have risks and can do harm if poorly designed and implemented [27, 30]. Specific potential harms to children and adolescents identified in the theoretical and empirical literature include heightened negative emotions including anger, distress and anxiety among those who are targets of racism, guilt and defensiveness among non-stigmatised groups, and increased stereotyping and bias and mistrust of other groups [27]. Programs that solely provide materials or are unstructured discussions have been found to be ineffective and likely to do harm [27, 31, 32]. Similarly, one off isolated events are likely to reinforce tokenistic, othering or celebratory approaches to diversity [27, 31, 32]. In contrast, interventions considered most effective are those that target structural, systemic and institutional change as well as individual-level attitudes and beliefs, and are grounded in developmental and theory and evidence [27, 31, 32]. This further reinforces the importance of empirically designed programs that have undergone high quality effectiveness evaluations to ensure interventions do no harm and do not perpetuate the very issues they are intending to address.

Schools are key settings in the lives of children and adolescents and become increasingly more important to identity formation as they develop and function outside of the family unit [33]. School communities, teachers and peers greatly influence children and adolescents beliefs about race and difference [34–36] and the extent to which they perpetuate, tolerate or intervene in situations of conflict [37]. Schools are also settings in which children and adolescents and their families experience and witness racism and racial discrimination [38–41]. This includes interpersonal discrimination from peers, teachers and other school community members as well as at an institutional, systemic level such as via curriculum content, pedagogical approaches and discipline procedures [42–44]. Schools are therefore key sites for the delivery of population-based programs to reduce racism and promote proactive bystander responses not only because they have “captive” populations but because they are powerful sources of influence on racism and responses to it at multiple levels.

While there is strong theoretical and empirical support for population-level school-based programs that counter racism and promote proactive responses to it, empirically tested programs are required. There remains an ongoing need for such programs to be evaluated rigorously, including their effectiveness in changing attitudes, beliefs and behaviours over time [32, 41, 45], their impacts on health outcomes [25] and any unintended negative consequences [27]. There are several theoretically sound, rigorously evaluated interventions that are shown to be effective at promoting prosocial bystander action in response to bullying and in reducing bullying behaviours [46–49] and at increasing the social norms within schools to reduce conflict [37]. Yet there is a lack of robust evidence for population-based school-based programs specific to racism and racial discrimination and associated bystander responses.

Racial discrimination and bullying are related but conceptually distinct factors in the lives of children and adolescents [12, 50, 51]. Racial discrimination is related primarily to group membership and social identities related to race and ethnicity, and fundamentally an expression of societal and structural forces of stratification and maintenance of status differences and social hierarchies [37, 50]. In contrast, bullying is related to individual traits and behaviours and occurs repeatedly at an interpersonal level [50]. Racism and racial discrimination interventions therefore need specific, theoretically driven components that address factors not involved in interpersonal bullying. Interventions focused on bullying are unlikely to be effective in addressing racism and racial discrimination if they do not address societal and structural drivers and forces at their core.

Best practice guidance for the development and evaluation of complex interventions, defined as interventions with several interacting components, recommends that they are developed using best available evidence and appropriate theory and then tested using a carefully phased approach starting with a series of pilot studies [52]. Pilot studies that assess feasibility and acceptability of the intervention are thus considered critical to the development and evaluation of complex interventions and provide valuable insights regarding the appropriateness of procedures, recruitment and retention, and acceptability of the program to participants. They also contribute important information about how the program can be modified before a more definitive effectiveness evaluation [52]. The use of mixed methods, both quantitative and qualitative, is considered particularly important in feasibility pilot studies [52]. Consistent with these recommendations, this study aimed to investigate the feasibility and acceptability of ‘Speak Out Against Racism’ (SOAR) a novel, multi-component intervention program to promote proactive bystander responses to racism and racial discrimination in Australian primary schools. Specifically, this study aimed to address the following research questions: 1) What was the effect of the SOAR program on: student reported bystander responses, self-efficacy to intervene, peer pro-social norms, and perceived school climate (primary outcomes); and student social and emotional wellbeing, school connectedness, sleep, and experiences of racial discrimination (secondary outcomes); 2) How appropriate were the survey measures for evaluation of this program?; 3) What were the experiences of school students and staff participating in the program?

### Overview of the SOAR program

The SOAR program was designed to be a whole of school, multi-level, multi-strategy program that aimed to promote effective bystander responses to racism and racial discrimination in primary schools. Specifically, it aimed to increase knowledge and practical skills for proactive bystander responses to racism and racial discrimination and to improve peer social norms and the school climate regarding racism and racial discrimination. SOAR drew on a multifaceted theoretical background, spanning theory and evidence on anti-racism, anti-bullying, prejudice reduction, child socio-cognitive development and social conflict in schools. This included evidence that the most effective programs are those that are: multi-level, considering stigmatised and non-stigmatised groups across intrapersonal, interpersonal, and systemic levels and include all students, those who experience racism as well as those who do not [27, 31, 53]; whole of school approaches that include school policies and guidelines, curriculum and pedagogy, teacher training and development, student support and development, parent and community involvement and monitoring and reporting [54]; focused on age-related cognitive skills and processing such as perspective taking and empathy, multiple classification, dual identity, moral reasoning (thinking and feeling about fairness) not only on intergroup contact [32, 41]; increase school prosocial norms to reduce conflict [37]; and sustained and integrated over extended periods of time [54].

SOAR spanned six mutually reinforcing elements: teacher training and development; curriculum and classroom materials; student support and development; parent and community involvement; school policies and guidelines; and monitoring and reporting of racial discrimination. *Teacher training and development:* 2 days of face-to-face training were provided for classroom teachers delivering the SOAR curriculum and classroom materials as well as any other interested school staff. These training workshops were presented by research staff from the Australian National University (NP, MT) together with staff from a service provider working with schools to support students of refugee backgrounds. New South Wales (NSW) Department of Education staff attended the NSW training and development workshops. After the training workshops, coaching sessions were delivered via phone or online throughout the program to enable staff to debrief with the research team and troubleshoot any issues. *Curriculum and classroom materials:* the SOAR classroom materials constituted an eight-week unit of work that included suggested activities and questions. The framework for this unit of work and classroom materials were developed by the research team (NP, OA), then workshopped and refined prior to implementation with classroom teachers and school support staff who had experience working with children from culturally, racially and religiously diverse communities, and reviewed by education department staff. These activities were facilitated in-class by classroom teachers, and culminated in the development of a class charter on pro-active anti-racist bystander action. *Student support and development:* the pedagogical approach of the SOAR program had a strong focus on students as experts and engaged learners throughout. This was foundational to the classroom materials and culminated in ‘Team SOAR’ student-led sessions for the second half of the program. ‘Team SOAR’ was a team of student influencers selected by teachers and classmates at the end of the eight-week unit of work to continue to promote the principles of SOAR. They held student-led meetings to develop materials and activities for their peers and the wider school community. *Parent and community involvement:* Team SOAR students developed activities to engage with parents and the wider community, and to communicate SOAR principles more broadly. *School policies and guidelines:* An audit tool was provided to support school leadership to review policies and guidelines regarding racism and racial discrimination and to develop a plan for future action in this area. *Monitoring and reporting of racial discrimination:* Schools were provided with the opportunity for school-level reports on survey data and the audit tool encouraged schools to review their monitoring and reporting systems, policies and guidelines.

## Method

### Study design and approach

A mixed-methods, quasi-experimental design was used to evaluate the SOAR program pilot. Quantitative data collection examined program effects via student surveys pre- and post- intervention and also enabled exploration of the appropriateness of these survey measures to evaluation of this program. The primary outcomes of the student survey were: student reported bystander responses, self-efficacy to intervene, peer pro-social norms, and perceived school climate. Secondary outcomes were: student social and emotional wellbeing, school connectedness, sleep, and experiences of racial discrimination. Quantitative longitudinal measures of change were collected via student questionnaires administered at baseline and follow up. Qualitative data included interviews and focus group discussions exploring experiences of school students and staff participating in the program and student and staff views regarding perceived need for the program, program implementation, program impacts and suggested improvements. The study was pre-registered with the Australian New Zealand Clinical Trials Registry (ACTRN12617000880347).

### Recruitment

To recruit schools, emails describing the SOAR program were sent in late 2017 to primary schools in two Australian states (New South Wales (NSW) and Victoria) and information about the study was disseminated via community and education networks. This information described the goals and content of SOAR and included details about how to join the study. Four volunteer schools (two in NSW and two in Victoria) agreed to participate in the study as intervention schools. Two volunteer schools in NSW agreed to participate as comparison schools. A further volunteer school in Victoria initially agreed to participate in the study as an intervention school but withdrew after classroom teachers became aware of the time commitment involved. Data from this school is not included in this present study. All students in Years 5 and 6 (ages 10–12 years) in each of the six participating schools were invited to participate in the study. Ethics approval was obtained from the Australian National University and from each state government education department, with permission obtained from each participating school principal and staff member alongside parent consent and student assent.

Quantitative data collection took place at the beginning of Term 1 in February 2018 and again at the end of Term 2 in August and September 2018 after completion of the SOAR program, in both intervention and comparison schools. (In Australia school starts late January and concludes mid-late December with the year broken into four terms). Students completed online or paper surveys in classrooms supported by SOAR research staff. Each school negotiated their preferred survey mode (online or paper) prior to data collection and all students in each school completed surveys using the same mode. Six hundred and forty-five students completed surveys across the six participating schools, 252 students in comparison schools and 393 students in intervention schools. Survey instruments had previously been used in a large scale population level survey of school students (*n* = 4664) across NSW and Victoria [11, 22].

Qualitative data collection took place in intervention schools in August and September 2018. Key informant interviews with school staff and focus groups with students were conducted. Ten staff interviews were conducted across the four intervention schools (five in each state). Those interviewed included teachers responsible for delivering the SOAR program, school leaders such as Assistant Principals, affiliated staff such as Anti-Racism Contact Officers and wellbeing teachers. Nine focus groups with students (five in NSW and four in Victoria) were conducted across the four intervention schools. Focus groups ranged from two to eight participants, with most containing six participants and ranged from 18 to 25 min duration. All qualitative data collection occurred on site at the participating schools and was audio-recorded and transcribed.

### Measures

#### Quantitative data

##### Primary outcomes

***Bystander responses***

Students were asked 11 items about their bystander responses adapted from the Participant Role Questionnaire [55]. Items comprised three sub-scales ‘Assistant’, ‘Defender’ and ‘Outsider’. Students were asked when they saw other students treated unfairly because of their race/ethnicity/cultural background how often they engaged in each response (e.g. I joined in, I helped the student being treated badly, I didn’t do anything). Response options were Never/Hardly ever/Sometimes/Most of the time/Always and scored 1–5. Sum scores of items were created for each scale, with high scores indicating a negative outcome for assistant and outsider scales and a positive outcome for defender scales (Cronbach’s α = 0.80 assistant; defender α = 0.90; outsider α = 0.74).

***Self-efficacy to intervene***

Students were asked four items about their self-efficacy to intervene when they saw other students treated unfairly because of their race/ethnicity/cultural background. They were asked ‘How confident are you that you could… stop it; help them feel better or cheer them up; go to a teacher for help; go to another adult for help.’ Response options were Not at all confident/Not very confident/Neither confident nor unconfident/Confident/Very confident and scored 1–5. A sum score of these items was created with a high score indicating a positive outcome [56–58] (Cronbach’s α = 0.80).

***Perceived school inter-ethnic climate***

Students were asked seven questions about their perceptions of the inter-ethnic climate of their school adapted from the School Interracial Climate Scale [59]. Three questions were about the teacher climate (e.g. Teachers encourage students to make friends with students of different racial/ethnic/cultural backgrounds; Teachers here like students of different ethnic groups to understand each other) and four about the peer climate (e.g. Students are able to make friends with students from different racial/ethnic/cultural backgrounds). Response options were Strongly disagree/Disagree/Neither agree or disagree/Agree/Strongly agree scored as 1–5. A sum score was created each for teacher and peer climate with a high score indicating a more positive climate (Cronbach’s α = 0.76 teacher; Cronbach’s α = 0.46 peer). Attempts to revise the peer climate scale and reduce the number of items using factor analysis did not increase the Cronbach’s alpha to an acceptable level.

***Peer pro-social norms***

Students responded to five questions about their perceptions of the pro-social norms of their school peers. They were asked ‘How many students at your school… Stand up for students who are made fun of or bullied?; Help other students even if they don’t know them well?; Care about other people’s feelings?; Help stop arguments between other students?; Are nice to everyone, not just their friends?’. Response options were Hardly any/Few/Some/Most/Almost all and scored as 1–5. A sum score of these items was created with a high score indicating a positive outcome [60] (Cronbach’s α = 0.87).

##### Secondary outcomes

***Socioemotional adjustment***

The Strengths and Difficulties Questionnaire (SDQ) is a brief questionnaire assessing the psychological adjustment of children and youth [61]. The youth-reported (11–17 years) SDQ consists of 25 items across five subscales. We examined total difficulties scale and child strengths in relation to prosocial behavior. The SDQ prosocial scale items reflect student perception of their own prosocial behaviour (I try to be nice to other people, I usually share with others, I am helpful if someone is hurt, I am kind to younger children, I often volunteer to help others). The SDQ is not intended to be used as a diagnostic instrument; rather, it indicates problematic emotions and behaviors across a range from normative to highly elevated [62]. While cut-points have been developed for the SDQ these have not been validated for ethnic minority youth; continuous scores are analyzed herein. The SDQ is the most commonly used measure of child and youth mental health in Australia and has been shown to be psychometrically sound in Australia [63, 64].

***School connectedness***

*Loneliness at school*

Five items asked students about their connectedness at school [65], e.g. ‘I have nobody to talk to; It’s easy for me to make new friends; I can find a friend when I need one’. Response options were Not true at all/Hardly ever true/Sometimes true/True most of the time/True all of the time coded as 1–5. Items were reverse coded as required and a sum score was created with a high score indicating a negative outcome (Cronbach’s α = 0.70).

*Teacher empathy*

Students were asked four questions about their teachers’ empathy adapted from the Department of Education and Training Victoria’s Attitudes to School Survey. e.g. ‘My teachers care about me; My teachers are good at dealing with racism when it happens’. Response options were Strongly disagree/Disagree/Neither agree nor disagree/Agree/Strongly agree. A sum score was created with a high score indicating a positive outcome (Cronbach’s α = 0.82).

*Inter-ethnic contact*

Five questions were asked about student inter-ethnic contact, e.g. ‘I have participated in cultural events with people from other racial/ethnic/cultural backgrounds’ [60] adapted from [66]. Response options were Strongly disagree/Disagree/Neither agree nor disagree/Agree/Strongly agree and scored as 1–5. A sum score of these items was created with a high score indicating a positive outcome (Cronbach’s α = 0.81).

***Sleep***

*Sleep duration* was self-reported with students asked what time they fall asleep and wake up on a usual school day and on a non-school day. Sleep duration was calculated as the difference between reported sleep time and reported wake-up time, separately for school and non-school days. *Sleep latency* was measured using a single item ‘During the last four weeks, how long did it usually take for you to fall asleep’. A 3-category analytic variable was created: 0–30, 30–60, > 60 mins [67]. *Sleep disruption* was measured using a single item ‘During the past four weeks, how often did you awaken during your sleep time and have trouble falling back to sleep again?’ A 3-category analytic variable was created: None/A little, Some/A good bit, Most/All. These items have previously been used with children and adolescents from diverse ethnic backgrounds [67].

***Racial discrimination***

*Direct experiences of racial discrimination*

Student reports of racial discrimination experiences were measured using 10 items drawn from the Adolescent Discrimination Distress Index [68] together with two items used previously with diverse Australian school students [39]; see Additional file 1. Items assessed discrimination by peers at school (4 items), by school personnel (3 items) and by others in the society (5 items). Each discrimination item was then followed by the attribution (‘because of…’) with ‘your race/ethnicity/cultural background’ being one of three non-mutually exclusive options. ‘Culture’ is commonly used to refer to race or ethnicity in Australian community vernacular so was included in the attribution following previous approaches [39, 51]. Frequency of each experience was indicated from 0=‘This did not happen to me’, 1=‘Once or twice’, 2=‘Every few weeks’, 3=‘About once a week’, to 4=‘Several times a week or more’. Following previous approaches [68] a sum score was created by calculating the mean frequency rating these items (Cronbach’s α = 0.91).

*Vicarious racial discrimination*

Student reported vicarious discrimination was measured using five items drawn from previous Australian studies; four items assessed discrimination from peers at school and one item assessed discrimination from teachers [39]. Students were asked how often they had seen other students treated unfairly e.g. treated with less respect by other students because of their race/ethnicity/cultural background (4 items), and how often they had seen ‘other students being picked on or treated with less respect by teachers at this school because of their race/ethnicity/cultural background?’ (1 item). Response options were 0 = Never, 1 = Hardly ever, 2 = Sometimes, 3 = Most of the time, and 4 = Always. Following previous approaches [68] a sum score was created by calculating the mean frequency rating these items (Cronbach’s α = 0.88).

##### Socio-demographics

Ethnicity was measured using a self-reported variable with categories developed for the study. Self-reported race/ethnicity is not routinely collected in Australia so a standard classification is not available. Students were able to select multiple categories and an open-ended ‘other’ category was available. Following international approaches [69], a prioritization method was used to classify multiple responses into mutually exclusive categories based on level of stigmatization in Australia in the following order: Indigenous; Pacific Islander, Maori, Middle Eastern, African, Latinx (non-Asian ethnic minority); Asian; Anglo/European (white) [22]. A ‘Other/missing’ category was also included in the ethnicity analytic variable. We recognize the considerable heterogeneity within each of these groups and do not wish to confuse or conflate Indigenous or ethnic identities while creating analytic categories with sufficient numbers for analysis. Religion was self-reported with the following analytic categories used (no religion, Christian, Islam, other religion (Buddhism, Hinduism, Judaism, other). Gender (female, male, and other) and School Year (5 or 6) were self-reported. Socioeconomic position at the school level was measured using the Index of Community Socio-Educational Advantage, a composite of average parent occupation and education across students, school geographic location, and proportion of Indigenous students [70].

Across the sample of *n* = 645 students, 15% had data available for baseline only; 11% had follow up data only. These missing data are likely related to student absenteeism on data collection days given the surveys were completed as a one-off during classroom time. Across the n = 645 sample, bystander response measures had the highest levels of missingness (10% no data) with other outcomes having far less missing (e.g. self-efficacy to intervene 2%; peer prosocial norms 4%; school climate 1%; discrimination 1%).

#### Qualitative data

Qualitative data collection entailed semi-structured interviews with classroom teachers and school leaders and semi-structured focus groups with students. Interviews and focus groups were guided by topic guides that explored staff and student experiences of the program including perceived need for the program, program implementation, program impacts and suggested improvements. Open-ended questions aimed to capture in-depth descriptions of the experiences and impacts of the project on staff, students and schools.

### Data analysis

#### Quantitative

Demographic characteristics of the sample by individual school and by study arm (comparison and intervention) were described. Multilevel mixed effect models were used for analysis of pre- and post- survey findings to account for the non-independence in the data, in our case paired data on individual students within a school. These models also enabled the analysis to make use of all data available, that is participants with baseline or follow up data only as well as those with data at both timepoints. A linear mixed model was fitted for each of the continuous outcomes and a logistic mixed model was fitted for each of the categorical outcomes. This study is both a pre-post study design and control/ intervention study, therefore in the models an interaction term is included. The interaction term is included in the model to quantify the effect of the intervention, as this represents the difference between study group (intervention compared to control) and the time period (post vs pre). A statistical difference is quantified as a *p* value of less than 0.05.

#### Qualitative

Following transcription of the recordings, interviews and focus groups were thematically coded and analysed using NVivo. The coding framework was developed using a combination of theoretically-driven (a priori) and inductively from the data. The data was analysed under ten thematic nodes. Staff and student data were analysed separately. Multiple researchers conducted coding and discussed findings iteratively with each other during the analysis process, as well as with the wider research team, to triangulate findings, enhance reflexivity and reduce the risk of bias.

## Results

### Characteristics of the study population

The participating schools spanned metropolitan, outer metropolitan and regional areas across NSW and Victoria. School characteristics and demographic characteristics of participating students by school and by intervention and comparison group are shown in Table 1. A total of 252 students completed surveys across the two comparison schools and 393 students across the four intervention schools. Student demographic characteristics by school, and by intervention and comparison group are shown in Table 1. Gender and year level were relatively balanced across all schools, and across intervention and comparison groups. Similar proportions of students identified as Indigenous across comparison (6%) and intervention (6%) schools, although these students were concentrated in schools and not evenly distributed across schools in the intervention group and the comparison group. In contrast, only 5% of students in the comparison schools identified as Middle Eastern, African, Pacific Islander or Latin American, compared with 13% in intervention schools; and 32% in comparison schools identified as Asian compared with 14% in intervention schools. In comparison schools, 36% of students identified with no religion, 9% identified with other religions, 2% identified their religion as Islam, and 53% identified their religion as Christian. In the intervention schools, 48% of students identified with no religion, 32% identified their religion as Christian, 10% identified their religion as Islam, and 10% identified with other religions.

**Table 1:** Student survey (*n* = 645) demographics by intervention and comparison group and by school

	Total (***n*** = 645)	Comparison			Intervention				
		School A (***n*** = 81)	School B (***n*** = 171)	Total (***n*** = 252)	Vic school A (***n*** = 108)	Vic school B (***n*** = 111)	NSW school A (***n*** = 30)	NSW school B (***n*** = 144)	Total (***n*** = 393)
**School characteristics**									
Socioeducational position (ICSEA)	–	914	1177	–	968	1104	924	937	–
Students in lowest quarter of socioeducational position (%)	–	47	1	–	45	7	56	47	–
Total enrolments	–	668	732	–	469	282	196	693	–
Indigenous students (%)	–	32	0	–	0	1	9	17	–
Language background other than English students (%)	–	7	55	–	70	14	74	4	–
**Student characteristics**									
Gender									
Female	332 (51.5%)	46 (56.8%)	81 (47.4%)	127 (50.4%)	55 (50.9%)	52 (46.8%)	15 (50.0%)	83 (57.6%)	205 (52.2%)
Male	310 (48.1%)	35 (43.2%)	88 (51.5%)	123 (48.8%)	53 (49.1%)	59 (53.2%)	15 (50.0%)	60 (41.7%)	187 (47.6%)
School year									
5	318 (49.4%)	41 (50.6%)	88 (51.8%)	129 (51.4%)	47 (43.5%)	63 (56.8%)	9 (30.0%)	70 (48.6%)	189 (48.1%)
6	326 (50.6%)	40 (49.4%)	82 (48.2%)	122 (48.6%)	61 (56.5%)	48 (43.2%)	21 (70.0%)	74 (51.4%)	204 (51.9%)
Ethnicity									
Indigenous	40 (6.2%)	15 (18.5%)	0 (0.0%)	15 (6.0%)	0 (0.0%)	5 (4.5%)	3 (10.0%)	17 (11.8%)	25 (6.4%)
Middle Eastern, African, Pacific Islander, Latin American	62 (9.6%)	2 (2.5%)	10 (5.8%)	12 (4.8%)	32 (29.6%)	6 (5.4%)	10 (33.3%)	2 (1.4%)	50 (12.7%)
Asian	135 (20.9%)	10 (12.3%)	70 (40.9%)	80 (31.7%)	38 (35.2%)	4 (3.6%)	7 (23.3%)	6 (4.2%)	55 (14.0%)
Anglo/Euro	339 (52.6%)	42 (51.9%)	78 (45.6%)	120 (47.6%)	27 (25.0%)	82 (73.9%)	6 (20.0%)	104 (72.2%)	219 (55.7%)
Other/missing	69 (10.7%)	12 (14.8%)	13 (7.6%)	25 (9.9%)	11 (10.2%)	14 (12.6%)	4 (13.3%)	15 (10.4%)	44 (11.2%)
Country of birth									
Australia	523 (82.0%)	72 (88.9%)	133 (80.1%)	205 (83.0%)	66 (61.7%)	100 (90.9%)	14 (46.7%)	138 (95.8%)	318 (81.3%)
Overseas	115 (18.0%)	9 (11.1%)	33 (19.9%)	42 (17.0%)	41 (38.3%)	10 (9.1%)	16 (53.3%)	< 10	73 (18.7%)
Religion									
Christian	253 (39.8%)	34 (43.6%)	97 (57.1%)	131 (52.8%)	24 (22.4%)	25 (23.4%)	12 (40.0%)	61 (42.7%)	122 (31.5%)
Islam	44 (6.9%)	2 (2.6%)	3 (1.8%)	5 (2.0%)	24 (22.4%)	0 (0.0%)	14 (46.7%)	1 (0.7%)	39 (10.1%)
Other religion	62 (9.8%)	8 (10.3%)	15 (8.8%)	23 (9.3%)	31 (29.0%)	3 (2.8%)	2 (6.7%)	3 (2.1%)	39 (10.1%)
No religion	276 (43.5%)	34 (43.6%)	55 (32.4%)	89 (35.9%)	28 (26.2%)	79 (73.8%)	2 (6.7%)	78 (54.5%)	187 (48.3%)

*NSW* New South Wales; *Vic* Victoria

### Quantitative findings

Results comparing study outcomes pre- and post-intervention across comparison and intervention groups are shown in Table 2. For the primary outcome of school inter-ethnic climate there was evidence of positive change. The intervention group had an increased student rating of the teacher interethnic climate at follow-up, while student rating of the teacher inter-ethnic climate did not change in the comparison group compared with baseline. There was no evidence that the change between pre and post scores for the other primary outcomes differed between the comparison and intervention group.

**Table 2:** Primary and secondary outcomes pre- and post- intervention by intervention group

Outcome^**a**^	Comparison		Intervention		B coefficient (95% CI) for interaction between intervention and time	***P*** value
	Pre (***n*** = 252) mean (SD)	Post (n = 252) mean (SD)	Pre (***n*** = 393) mean (SD)	Post (***n*** = 393) mean (SD)		
Primary outcomes						
Bystander responses						
Assistant (2–10)	2.3 (1.2)	2.3 (0.8)	2.5 (1.4)	2.4 (1.1)	− 0.04 (− 0.34, 0.26)	0.8
Defender (7–35)	24.6 (8.1)	23.6 (8.3)	26.4 (7.2)	26.1 (7.4)	0.41 (−1.12, 1.94)	0.6
Outsider (2–10)	4.2 (2.0)	4.3 (1.9)	4.0 (2.1)	4.2 (2.0)	0.09 (− 0.35, 0.53)	0.69
Prosocial norms and self-efficacy						
Prosocial norms (2–20)	17.6 (4.4)	17.1 (4.3)	18.3 (4.4)	17.9 (4.4)	0.3 (−0.53, 1.14)	0.47
Self-efficacy to intervene (4–20)	15.9 (3.3)	15.6 (3.5)	16.5 (2.9)	16.5 (3.3)	0.31 (− 0.34, 0.96)	0.35
School inter-ethnic climate						
Peer inter-ethnic climate (4–20)	15.8 (2.9)	15.9 (2.9)	15.6 (2.8)	15.8 (2.7)	0.27 (−0.3, 0.83)	0.35
Teacher inter-ethnic climate (3–15)	11.5 (2.2)	11.5 (2.4)	11.9 (2.2)	12.4 (2.4)	0.5 (0.02, 0.98)	0.04
Secondary outcomes						
Socioemotional adjustment						
SDQ: total (0–40)	15.0 (4.3)	13.1 (5.1)	15.4 (4.7)	14.1 (5.2)	0.34 (− 0.49, 1.17)	0.42
SDQ: prosocial (0–10)	8.2 (1.7)	8.1 (1.6)	8.0 (1.7)	8.2 (1.7)	0.3 (0, 0.6)	0.05
School connectedness						
Teacher empathy (0–8)	7.2 (1.4)	7.0 (1.7)	7.4 (1.2)	7.2 (1.5)	−0.01 (− 0.3, 0.28)	0.96
Loneliness at school (5–25)	9.1 (3.7)	8.4 (3.4)	9.3 (3.4)	8.9 (3.4)	0.29 (− 0.32, 0.9)	0.36
Inter-ethnic contact at school (5–25)	19.5 (3.5)	20.2 (3.4)	19.6 (3.6)	20.6 (3.5)	0.29 (− 0.35, 0.93)	0.37
Sleep						
Sleep duration	574.9 (64.8)	577.5 (64.4)	580.0 (84.1)	580.6 (62.6)	11.93 (−5.75, 29.62)	0.19
Sleep latency						
0–30 min	110 (55.8%)	122 (64.6%)	212 (65.2%)	211 (68.5%)	0.23 (−0.46, 0.91)	0.52
30–60 min	58 (29.4%)	41 (21.7%)	71 (21.8%)	57 (18.5%)	–	–
> 60 min	29 (14.7%)	26 (13.8%)	42 (12.9%)	40 (13.0%)	–	–
Sleep difficulties						
Mild (a little of the time/none of the time)	31 (15.5%)	25 (13.2%)	67 (20.6%)	43 (13.8%)	−0.05 (− 0.64, 0.54)	0.87
Moderate (a good bit of the time/some of the time)	60 (30.0%)	41 (21.7%)	82 (25.2%)	76 (24.4%)	–	–
Sleep problem (all of the time/most of the time)	109 (54.5%)	123 (65.1%)	177 (54.3%)	193 (61.9%)	–	–
Racial discrimination						
Direct (0–48)	1.5 (4.1)	1.2 (3.1)	1.8 (4.8)	1.4 (4.0)	−0.05 (−0.89, 0.79)	0.9
Vicarious (0–20)	4.3 (4.2)	3.9 (3.6)	3.6 (4.0)	3.1 (3.9)	−0.22 (− 0.91, 0.46)	0.52

– = not applicable; *CI* confidence interval; *NSW* New South Wales; *SD* standard deviation; *SDQ* Strengths and Difficulties Questionnaire; *Vic* Victoria

^a^Possible score ranges for each scale denoted in brackets

For the secondary outcomes there was evidence of change in the students’ prosocial score in the intervention group compared with the comparison group. The intervention group had an increased SDQ prosocial score at follow-up, while those in the comparison group had a reduced SDQ prosocial score at follow-up, compared with baseline. There was no evidence of an interaction between group (intervention or control) and the pre-post time period on the remainder of the outcomes, including racial discrimination. That is, there was no evidence that the intervention did harm in terms of increasing racial discrimination, or increasing mental health difficulties or sleep difficulties.

### Qualitative findings

Qualitative data presented below addresses the following broad themes: SOAR addresses a key gap in proactive programs addressing racism; initial apprehensions about the program overcome; program impacts; and limitations and improvements in the SOAR program.

#### SOAR addresses a key gap in proactive programs addressing racism

School staff discussed how SOAR addressed a key gap in the curriculum and in school activities, in being a proactive program addressing racism among students as well as staff.

*In the past we probably hadn’t done a lot of sort of proactive you know activities, programs, it was more just reacting to kids with issues and trying to sort them out (Victoria School A, Interview 3, School Leader).*

*But there’s probably nothing directly in the curriculum about racism as such…there’s a million things we could be doing but I think it’s probably really important to have those discussions (Victoria School B, Interview 4, Teacher).*

*But it’s not so much the children, it’s probably more so some of the staff (NSW School B, Interview 1, Teacher).*

Students also discussed that parents in their school community had been supportive of the program going ahead and saw it as meeting a need both at their school as well as in wider society.

*I mentioned it to my parents and they thought it was a good idea that we’re learning about it and thinking about it, it’s not just in our school, it’s happening all around the world and in public areas and sporting events and everywhere (NSW School A, Focus Group 3, Student).*

#### Initial apprehensions overcome - SOAR was fun, engaging, well-structured and scaffolded, supporting students as expert learners

Overall, students and staff were very positive about the SOAR program despite some initial apprehensions.

Students described appreciation for having opportunities to write down their *‘own opinion’* (Victoria School A, Focus Group 1, Student) and many indicated that they enjoyed the way SOAR enabled them to contribute their thoughts and ideas.

Teachers also expressed appreciation for the well-structured and scaffolded nature of the SOAR program and classroom activities.

*The lessons were great and you know, the way we sort of introduced it at the very beginning was really good too, like you know, the whole, yeah, just some of those activities were really good and it generated really good discussion with our kids across all classes (NSW School A, Interview 2, School Leader).*

*The fact that there were some resources provided for the teachers, some structured lessons, things like that that’s given them a platform to yeah raise the topic in class with a little bit of credibility whereas some people might feel like oh I’m not sure what to say or I don’t know how to broach this subject (Victoria School A, Interview 3, School Leader).*

Students in one focus group highlighted that some students responded to the program activities with laughter and discussed the potential impact of this on students experiencing racism. This reinforces the need for ongoing support for teachers delivering the program in both managing students’ discomfort and distraction behaviours while also supporting students experiencing racism themselves.

*There were some people that like might as well – like they didn’t get it sort of and they would like make fun and start laughing and stuff and it was like in the dark point and they thought it was funny and everything when there probably are other students that might maybe be feeling that way so that could hurt them at the same time… (Victoria School A, Focus Group 1, Student).*

Students and staff spoke enthusiastically about the ‘Team SOAR’ activities, describing how the students mobilised and found conviction, satisfaction and wider identification within and outside their school contexts.

*The ideas have just flown about how to get the word out and about how to develop the team so my – the kids have just been – just inundating with ideas, it’s been amazing. They really want to – brought out the ‘SOAR patrol’ logos and that sort of stuff and say why they represent racism and you know or against racism, I should say, how they would get the message out through the school and how about going through the community and that sort of stuff like community radio (Victoria School A, Interview 2, Teacher).*

*So, the kids took it on and then we decided that every child, every teacher, and every parent that was around would get one of these bracelets, so then it madly went from the SOAR team to ending up having 40 and 50 children coming in at a recess and lunchtime making them. … I mean it spread madly throughout the school, throughout the community and then we had an assembly where we spoke about what the SOAR program was (NSW School A, Interview 1, Teacher).*

*It was so good because when we came back out people were saying like good job and like clapping and when they put it on Facebook everyone all around the community saw it and they were moved by it and our teacher said that she knew we had done a good job because of that (NSW School B, Focus Group 2, Student).*

#### Program impacts

Overall, staff and students were very positive about the impacts of SOAR on teachers, students and the school community.

*You’re upskilling teachers to deal with racism as an issue and then you’re empowering children to be drivers of the cause (Victoria School A, Interview 3, School Leader).*

##### Teacher attitudinal and behaviour change

Some staff discussed the impact of the teacher training and the program on their own attitudes and behaviours regarding racism and discrimination both at school and in the wider community.

*I think there was a big impact really for the teachers because we were having discussions and you know, we probably shouldn’t say things that way or we should probably rethink how we do that and just incidental things of you know, how we talk about different things (NSW School A, Interview 2, School Leader)*

*Yeah, I sort of appreciate it more when I’m sort of out and about and see it like on the weekends and that sort of stuff. And now that I know sort of not to be a bystander and step in … But yeah now I think it’s more – for me personally it’s just a mind-opener (Victoria School A, Interview 2, Teacher).*

However, as illustrated by these quotations, discussions of racism among school staff remained focused on interpersonal racism without mention of structural and institutional racism.

##### Improved student prosocial norms and school climate, reduced racial discrimination

Students and teachers discussed changes to peer prosocial norms, school climate and experiences of racial discrimination resulting from SOAR.

*Student: Everybody is nicer to everyone, yeah, they’re treating them equally but before the SOAR program people are just pushing each other and like fighting but then after the SOAR program they just learned that everyone has their own rights and that…*

*Student: Everyone is an equal (NSW School B, Focus Group 2, Student).*

*I think people have definitely like taken away from it positively because they’ve been like not joking about things like this anymore or like a lot of immature people have not been so ignorant or just one-minded (NSW School A, Focus Group 3, Student).*

*Doing playground duties, like you can definitely see just the different interactions of kids in terms of issues happening, a lot of them seem to be a bit more involved positively to try and fix it which was cool to see (NSW School A, Interview 1, Teacher).*

Some students discussed that they felt these prosocial norm changes extended beyond the school to their interactions in the wider community.

*I think that children at school not just feel safer about accepting other people but like beyond the school grounds like someone in a shop, someone down the road that you’re just going for a daily walk just to smile at them and not to steer away (Victoria School B, Focus Group 4, Student).*

However, the extent to which observed changes continued beyond the SOAR program were raised by some students.

*I just felt like when we were doing SOAR it was sort of better out there but since we finished there was more things that were going bad so I feel like it was like we finished it, we moved on and I feel like people forgot about it (Victoria School A, Focus Group 1, Student).*

While most students described positive changes resulting from SOAR, some discussed that they felt there had been little change. Students suggested this was due to both the lack of cultural diversity and the high level of cultural diversity in their respective schools.

*I haven’t noticed any difference because our school isn’t actually very culturally diverse – like it’s really not like I don’t think there’s much that you can stand up for (Victoria School A, Focus group 3, Student).*

*There’s not much racism ‘cause there’s so much like cultures around (Victoria School A, Focus Group 2, Student).*

##### Student knowledge of proactive bystander responses, confidence and self-efficacy to intervene to address racism

Staff and students discussed how they believed SOAR had increased student knowledge regarding potential bystander responses to racism*.* Students reflected on the value of practical steps they could take to respond to racism.

*Student: Like you could go over and comfort and ask them if they wanted to play the game you’re playing…*

*Student: You could confront the bully and say, or the racist and say I don’t like what you said or something like that, if you’re brave enough to do that.*

*Student: Yeah, only if you’re confident enough.*

*Student: Also, you could tell a parent or any other responsible adult that you know is around.*

*Student: Like if it happens on like public transport and you’re going home on a bus or something you can always tell like the bus driver or a parent (NSW School A, Focus Group 3, Student).*

*Just gave us more like solutions, just like kind of small situations that we could kind of – just in our own way we could kind of just like stop it... it just showed us way we could deal with it in our lives, not just the whole big thing of racism sucks and we need to get rid of it (Victoria School B, Focus Group 3, Student).*

*And we had an incident recently where we were able to bring up what a bystander issue again and reflect on it. We had a computer messenger video incident, we were able to talk about what a bystander was again and the kids knew and even though they weren’t a successful bystander on this occasion they were able to reflect and know what that is and know what they should do next time so I think that’s really important because it’s inevitable that’s going to happen regularly (Victoria School B, Interview 5, Teacher).*

Some students also described how SOAR had helped them feel more confident in relation to addressing racism as well as more broadly.

*It can make them feel more confident to stand up for people (Victoria School B, Focus Group 3, Student).*

*And, also, like what SOAR means, it’s like speak out against racism which I think it’s a really good like thing and like [other student] said, it like gets people to have more confident in their self and like just speak out and like just sort of have like an opinion and like tell them what’s actually happening (Victoria School A, Focus Group 1, Student).*

*it’s like that’s kind of made me come out of my shell and like made me more confident and more like – I feel more like brave, probably. Like I will start putting my hand up even when they’ll probably – like say maths or something (Victoria School A, Focus Group 1, Student).*

##### Increased awareness of racism and commitment to anti-racism

Students discussed how the program had increased their understandings and awareness of racism and its expressions, including concepts such as stereotypes.

*Student: It’s a fantastic program that should be used in each school and it’s great for like children to learn about and learn about all the races and the bullying that’s happening everywhere.Student: Yeah, I agree, it should be taken to other schools because if it’s happening a lot in other schools well then they’d be able to learn about it and realise that it’s not okay (NSW School A, Focus Group 3, Student).*

*Stereotype like I heard people like talking about stereotypes but I never really knew what it meant until we were talking about different types of stereotypes (Victoria School A, Focus Group 2, Student).*

This increased awareness of racism in the world was described as an inspiration to anti-racism action among students and a commitment to acceptance of difference and the right to fair treatment for all.

*It was really helpful like I definitely learnt a lot and I think it – there’s a lot of stuff that I kind of heard about that I didn’t – like I hadn’t really heard of before like things that are going on in our world that aren’t really okay and it kind of inspired me to want to do something about it (Victoria School B, Focus Group 3, Student).*

*Student: I think I've learned that it’s not okay to be bullied or, yeah, to be bullied just because you eat a different food to someone else or you have a different colour of skin or what you wear to someone else, we should all be treated the same because we’re all humans.*

*Student: I definitely think it’s very important because like as she said we are all humans and we all have rights to be ourselves and to be okay with that and nobody should be able to take it away. It’s a really great and comforting program for those that have a different ethnicity or race to know that they're being supported by the people (NSW School A, Focus Group 3, Student).*

*Student: That it’s okay to be different.*

*Student: Yeah.*

*Student: Yeah, and that everyone is different in their own ways that they can’t change what they are.*

*Student: And if everyone was the same it would be so boring (NSW School B, Focus Group 2, student).*

#### Limitations and improvements

An already crowded curriculum and demands on teacher workload were commonly discussed as limitations to program implementation.

*Time management is the thing, and to do it justice you can’t just do just a little bit here and a little bit there (NSW School B, Interview 1, Teacher).*

*…we don’t really kind of look into racism that much because as you’re probably aware the teaching curriculum is extremely overloaded. These poor teachers are just flat out and I’m not saying that as a sympathy-type thing, I’m saying that to be a realist. The amount of work that’s on these teachers is full-on (Victoria School B, Interview 5, Teacher).*

The need for school leadership support for the program was also reinforced by teachers as a key success factor.

*But the executive needs to be supportive of the whole school yes, so if you have someone that’s appreciative of the program and feels, well, there’s value behind it, if the people at the top see value in it then it’s easier to share with staff (NSW School B, Interview 2, School Leader).*

Suggested improvements for the program were: translating materials into languages other than English to support engagement from students who were from refugee or newly arrived immigrant backgrounds, providing refresher lesson materials to continue the ongoing presence and sustainability of the program in schools, further exploring ways to increase parent and community involvement, and greater support for connection between participating schools.

## Discussion

This study provides quantitative evidence of the potential of the SOAR program to improve the prosocial skills of primary school students and their perceptions of the inter-racial school climate provided by their teachers, and critically, to not cause harm. It also provides qualitative evidence of the SOAR program’s potential to promote teacher attitudinal and behaviour change regarding racism, to reduce interpersonal racial discrimination, and to improve peer prosocial norms, awareness of racism and commitment to anti-racism, knowledge of proactive bystander responses and confidence and self-efficacy to intervene to address racism among primary school students. Study findings address critical gaps in the current empirical evidence regarding population-level school-based programs to counter racism and promote proactive responses to it. The urgent need for robust empirical evidence supporting such programs, including their effectiveness in changing attitudes, beliefs and behaviours over time [32, 41, 45], their impacts on health outcomes [25] and any unintended negative consequences [27] are outstanding issues to which this study makes important and novel contributions.

The SOAR program is a whole of school, multi-level, multi-strategy program that moved beyond generic bystander bullying programs to specifically target racism. While there are several theoretically sound, rigorously evaluated interventions shown to be effective at promoting prosocial bystander action in response to bullying and in reducing bullying behaviours [46–49] and at increasing the social norms within schools to reduce conflict [37], there has been a lack of such evidence specific to racism and racial discrimination and associated bystander responses in schools. Addressing racial discrimination is considered to require specific strategies and a unique evidence base separate to bullying [45, 50]. Consistent with these recommendations, the SOAR program drew on a multi-faceted, multi-disciplinary theoretical background, spanning theory and evidence on anti-racism, anti-bullying, prejudice reduction, child socio-cognitive development and social conflict in schools [27, 31, 32, 37, 41, 53, 54]. It is also consistent with recent recommendations for such programs to be multi-component and multi-layered, to consider interpersonal and structural levels of intervention [45].

Study findings highlighted a number of areas for improvement in the intervention design. Suggestions for development and refinement of the program need to be taken into consideration for future implementation. These include exploring how best to resource the program within already high teacher workloads and existing curriculum content, for example providing additional funding to support teachers to participate in the program and backfill their other duties, releasing teachers from other commitments or linking the program more directly to existing curriculum content and deliverables, maximising school leadership engagement and support, supporting teachers to provide refresher lessons for students after the program’s completion, enabling translation into languages other than English and cultural adaptation more broadly, and enhancing parent and community involvement within participating schools, as well as collaboration between schools. Further qualitative and co-design work with school staff and with parents and community members to develop and refine the intervention and its implementation to ensure it is contextually appropriate and feasible is an important future step. Despite these identified improvements, overall the study findings are promising and a larger implementation trial of the program is recommended using methods such as a stepped-wedge cluster randomised trial [71]. Such a trial would allow testing of the program at scale, as well as provide a larger sample size to examine which groups of schools and students are most likely to benefit from the program, under what conditions, and for whom further modifications or targeted approaches may be required. This would include exploration of whether the program has different impacts for students from majority and minoritized backgrounds, and in what contexts. As appropriate in this initial feasibility pilot study, interested schools volunteered to participate in the program and the study. This has high potential to introduce selection bias, with schools already motivated or ready to change likely to be more willing to volunteer. The volunteer-based study recruitment also resulted in different socio-demographic profiles across intervention and comparison schools which may have influenced study findings. A larger implementation trial would assist to reduce these sources of bias.

In interpreting quantitative findings from this present study, it is important to note that baseline levels of outcome measures across groups were all at relatively positive levels before the intervention commenced, meaning that further improvement on these already positive levels was difficult to achieve. Assistant scores were particularly close to the lowest score possible, with defender scores showing a little more, but not a lot of, room for improvement. Overall, bystander response scores and reported racial discrimination scores were more positive than the state population levels reported using the same measures [11]. There were high levels (23–40%) of missing data in the bystander response measures, by far the highest of all of the survey measures included. This suggests that this may not be the most appropriate measure to capture bystander responses, and alternatives are needed for future studies. Developing and testing alternate measures is an area for future work. The peer inter-ethnic climate measure also did not perform well in this sample. Exploration of potential reasons for this and identification of alternative measures for this construct are needed appropriate to this population and context. A larger implementation trial would also benefit from a broader set of outcome measures across a wider range of constructs, as informed by the qualitative component of this current study. Ensuring survey instruments are short and age-appropriate to minimise response burden is also important. It is also highly plausible that the intervention had different effects for different ethnic groups. This was not able to be explored in this present study as it was not sufficiently powered to do so, but should be examined in future larger studies.

Qualitative findings identified student and teacher reported increases in primary school students’ self-efficacy to reduce interpersonal racial discrimination and to improve peer prosocial norms, as well as awareness of racism and commitment to anti-racism, knowledge of proactive bystander responses and confidence and self-efficacy to intervene to address racism. Using more specific and sensitive measures of these constructs in future implementation studies would be important to capture these changes not presently identified in the quantitative data from this study. Collection of quantitative data from teachers, including regarding their attitudes and knowledge regarding racism is also recommended for future implementation studies. Qualitative data regarding student and teacher experiences of the program remain important to future work, with further attention to data collection regarding the audit tool also needed, as this was not discussed by participants in this current study. Greater attention to collection of implementation data drawing on implementation science methods would also be helpful.

## Conclusion

This study provides quantitative evidence of the potential of the SOAR program to improve the prosocial skills of primary school students and their perceptions of the inter-racial school climate provided by their teachers. It also provides qualitative evidence of the SOAR program’s potential to promote teacher attitudinal and behaviour change regarding racism, and to reduce interpersonal racial discrimination, and to improve peer prosocial norms, awareness of racism and commitment to anti-racism, knowledge of proactive bystander responses and confidence and self-efficacy to intervene to address racism among primary school students. Further refinement and testing of the program in a large scale implementation trial is recommended. Addressing racism and racial discrimination must a core public health priority, even more so among children and young people. Developing, implementing and evaluating rigorous programs to counter racism and to teach all children and young people to recognise and reject racism are vitally important human rights and health equity goals for societies as a whole.

## Supplementary Information

**Additional file 1.**

## Data Availability

The datasets used and/or analysed during the current study available from the corresponding author on reasonable request.

## Abbreviations

NSW: New South Wales
SDQ: Strengths and Difficulties Questionnaire
SOAR: Speak Out Against Racism
