# Supplementary material for: Promoting proactive bystander responses to racism and racial discrimination in primary schools: a mixed methods evaluation of the ‘Speak Out Against Racism’ program pilot
Source: BMC Public Health. 2021 Jul 21;21:1434. doi: 10.1186/s12889-021-11469-2 (PMC8293475; doi:10.1186/s12889-021-11469-2)
Supplement: Supplementary file 1 — Additional file 1. [file 12889_2021_11469_MOESM1_ESM.pdf]

Appendix 1

Racial Discrimination

Below are some situations where other students said they were treated unfairly. We want to know if any of these things have EVER happened to you. These things could happen to you in person by another person or a group of people, or online, or using a mobile phone or the internet such as text messages, pictures, video clips, emails being sent to you or sent to others, about you.

a. Were you called insulting names by other students …

b. Did other students leave you out of their activities…

c. Did people think you didn't speak English well…

d. Did people tell you that you don't belong in Australia…

e. Did other students spit on you, push you or hit you…

f. Were you threatened by other students…

g. Did people act like they did not trust you…

h. Did you get poor service at a restaurant or fast food place (e.g. being ignored)…

i. Were you treated unfairly by a shop assistant or security guard…

j. Were you put in a lower ability class or group…

k. Were you disciplined unfairly or given school detention…

l. Were you given a lower grade or mark than you deserved…

Each item was followed by the attribution: "Because of your race/ethnicity/cultural background?" and students were given the following response options to choose from: 1) This did not happen to me, 2) Once or twice, 3) Every few weeks, 4) About once a week, 5) Several times a week or more.

Note: a, b, e, f peer sources of discrimination; j, k, l school sources of discrimination; c, d, g h, i societal sources of discrimination.
